# Sphingosine-1-phosphate receptor 1 signaling stimulates human pluripotent stem cell-derived cardiomyocyte differentiation and maturation

**DOI:** 10.1038/s12276-026-01767-3

**Published:** 2026-07-02

**Authors:** Won Dong Yu, Seul-Gi Lee, Kwang Bo Jung, Sung-Ae Hyun, Young-Dae Kim, Ye Seul Son, Hana Lee, Hyun-Soo Cho, Dae-Soo Kim, Kyung Jin Lee, Jongman Yoo, C-Yoon Kim, Mi-Ok Lee, Ohman Kwon, Mi-Young Son

**Affiliations:** 1 Korea Research Institute of Bioscience and Biotechnology (KRIBB), Daejeon, Republic of Korea; 2 KRIBB School of Bioscience, Korea University of Science and Technology (UST), Daejeon, Republic of Korea; 3 College of Veterinary Medicine, Konkuk University, Seoul, Republic of Korea; 4 Research Group for Safety Pharmacology, Korea Institute of Toxicology, KRICT, Daejeon, Republic of Korea; 5 R&D Institute, ORGANOIDSCIENCES, Seongnam, Republic of Korea; 6 CHA Organoid Research Center, CHA University, Seongnam, Republic of Korea; 7 School of Medicine, Sungkyunkwan University, Suwon, Republic of Korea

**Keywords:** Stem-cell differentiation, Induced pluripotent stem cells

## Abstract

The development of human-relevant in vitro cardiac models is essential for evaluating cardiotoxicity and drug efficacy. Although wingless-related integration site signaling-based protocols have enabled cardiomyocyte (CM) differentiation from pluripotent stem cells, issues such as low efficiency, immature phenotype, and variability across cell lines remain to be resolved. Here, we present an improved differentiation strategy that robustly enhances both the efficiency and functional maturity of CMs, comparable to the functional characteristics of human adult myocardial tissue. Although fetal bovine serum enhanced differentiation, we disclosed that the interaction between sphingosine-1-phosphate (S1P) and its receptor S1PR1 is a major contributor. Gene-based and protein-based analyses and electrophysiological analysis via multielectrode array confirmed that activation of S1PR1 using S1P or its agonist SEW2871 increased the generation of spontaneously beating CMs, whereas S1PR1-deficient pluripotent stem cells exhibited impaired differentiation and functional maturity of S1P-S1PR1-derived CMs. Furthermore, we observed improved cardiac function and reduced fibrosis in a myocardial infarction mouse model induced by left descending artery ligation following transplantation of CMs differentiated more robustly by treatment with S1P or SEW2871. Collectively, S1PR1 activator-treated CMs offer a cost-effective platform for drug discovery and regenerative medicine.

## Enhanced cardiac models boost drug discovery and regeneration

The critical role of heart in the circulatory system makes cardiac damage from chemotherapy or cardiovascular diseases a life-threatening concern. This study addresses the need for an in vitro cardiac cell model that accurately reflects human heart physiology. Researchers utilized human pluripotent stem cells to develop cardiomyocytes (CMs) with improved differentiation efficiency and maturity by activating the sphingosine-1-phosphate receptor 1 (S1PR1) signaling pathway. The study used gene expression analysis, immunostaining, and electrophysiological assays to demonstrate that S1P or its agonist SEW2871 significantly enhanced CM differentiation and functional maturation. These CMs exhibited increased cell area, multinucleation, and sarcomere length, indicating enhanced structural organization and functionality. The study’s findings suggest that S1PR1 activation could lead to more mature and functional human pluripotent stem cell-derived CMs, offering potential advancements in cardiac disease modeling, drug screening, and regenerative therapies.

This summary was initially drafted using artificial intelligence, then revised and fact-checked by the author.

## Introduction

The heart has a crucial role in the circulatory system and pumps the blood throughout the body. Hence, cardiac damage caused due to chemotherapeutic drugs or cardiovascular diseases is critical for determining the crossroads between life and death. Consequently, the development of in vitro cardiac cell model that reflects the human heart structure and function has been required for several decades. Because primary cardiac cells cannot be used owing to the absence of expandable stem cells in human heart tissues, human pluripotent stem cells (hPSCs), exhibiting the ability to differentiate into every cell type, including cardiomyocytes (CMs), are utilized to develop predictive models reflecting human heart physiology^1–6^.

Despite various proposed CM differentiation methods based on the cellular signaling pathway and mesodermal development process, further improvements are warranted for achieving the robustness of the differentiation protocol and increased cell purity with xenogenic-free chemically defined media. Hence, various adjusted soluble factors, including cytokines and extracellular matrix, have been explored to enhance mesodermal specification and cardiac differentiation^1,4,7–10^. For instance, bone morphogenetic protein can induce mesodermal differentiation resulting in the lineage specification into the CMs^1,8^. Similarly, various extracellular matrix components, such as fibronectin and laminin, can promote cardiac differentiation from hPSCs^10,11^. The chemically defined method of Burridge et al. is among the most used protocols to generate CMs^1^. Notably, cardiac differentiation initiates with the wingless-related integration site (WNT) signaling pathway activation, followed by the subsequent inhibition of its downstream molecules, which induces hPSC differentiation into mesodermal lineage cells. This method provides reproducible and scalable hPSC-derived CMs required for disease modeling or drug discovery; however, different key challenges persist, including CMs’ low maturation levels and variable differentiation efficiency across cell lines^7,8,12^.

Reportedly, transient treatment of the 3D embryoid body with fetal bovine serum (FBS) increases differentiation efficiency by upregulating both CM marker gene expression and functional properties^13^. Nonetheless, the higher complexity and heterogeneity with limited scalability of 3D cardiac models restrict the high-throughput screening of drug candidates or therapeutic applications. By contrast, simple, cost-effective, and homogeneous 2D CM differentiation methods exhibit high reliability, enabling high-throughput screening and standardized therapeutic applications despite CMs presenting immature phenotype and lack of physiological relevance. Therefore, developing a 2D PSC-derived cardiac model with greater maturity and functionality is crucial to expand the future applications of the cardiac models.

Although FBS treatment enhances the differentiation efficiency of PSC-derived CMs, consistent with our previous report, FBS use also presents several limitations for translational drug testing owing to batch-to-batch variability, xenogenic factors, and undefined composition. Therefore, we aimed to use a novel strategy to enhance hPSC-derived CM differentiation by elucidating the role of sphingosine-1-phosphate (S1P) signaling and its interaction with S1P receptor (S1PR)1, which can be activated by FBS treatment. S1P is a bioactive lipid mediator signaling molecule that regulates cellular behavior via G protein-coupled S1P receptors^14^. Sphingosine kinase (SphK)1 and SphK2 are involved in S1P biosynthesis and have distinct roles in cardiac development, protection, and regeneration^15–17^. The roles of S1P–S1PR1 signaling is well established in cardiovascular physiology, particularly in regulating ion channel activity and contractility^14,18,19^, but its specific contribution to CM differentiation and functional maturation remains elusive. Herein, the effect of activation of the S1P–S1PR1 axis on CM differentiation efficiency and maturity was determined through gene expression analysis, immunostaining, microarray profiling, and multielectrode array (MEA) assays. Furthermore, CRISPR-Cas9-mediated gene editing was performed to confirm the essential role of the S1P–S1PR1 interaction in this process. Finally, the therapeutic potential of S1P signaling was validated utilizing a myocardial infarction (MI) mouse model, showing the effects of S1P or its selective agonist SEW2871 on cardiac regeneration following left anterior descending (LAD) artery ligation.

## Methods

### Cell culture

The human embryonic stem cell (hESC) H9 line and human fibroblasts (CRL-2097 and IMR90) were purchased from the WiCell Research Institute (Madison, WI, USA) and the American Type Culture Collection, respectively. hPSCs and somatic cells were cultured as described previously^20^. Human-induced pluripotent stem cells (hiPSCs) were generated from human fibroblasts utilizing Episomal iPSC Reprogramming Vectors (Invitrogen, A14703, Carlsbad, CA, USA), and the generated hiPSC colonies were expanded and further characterized as described previously^20,21^. This study was approved by the Korean Public Institutional Review Board (IRB nos P01-201409-ES-01-09 and P01-201609-31-002).

### Differentiation of hPSCs into human CMs

Three different hPSC lines including hESC line (H9) and hiPSC lines (CRL-2097-hiPSC and IMR90-hiPSC) were used in a research study, and cells showing ideal cellular morphology were selectively detached and seeded into the 1% Geltrex-coated tissue culture plates containing mTeSR medium without feeder cells. For mesodermal induction, hPSCs were treated with 6 μM CHIR99021 (Tocris, 4423) in CM differentiation medium (CDM) containing RPMI1640 (Gibco, 11875119) supplemented with 212 μg/ml L-ascorbic acid (Sigma, A8960) and 500 μg/ml albumin (Sigma, A0237) for 2 days. After 48 h of mesoderm induction, cells were treated with 2 μM Wnt-C59 (Selleckchem, S7037) in CDM for another 2 days. Subsequently, the cells were cultured with CDM for 2 days and then replaced with CM maintenance medium, containing RPMI1640 supplemented with 212 μg/ml L-ascorbic acid, 1× B-27 (Gibco, 12587010), and 1% chemically defined lipid mixture (Thermo, 11905031). We defined these cells as “control CM”. To enhance the differentiation of PSC-derived CMs, FBS (Gibco, 26140079) was added for 2 days on differentiation days 0–2. Alternatively, 0.1 μM S1P (Tocris, 1370), 0.5 μM SEW2871 (Tocris, 2284), or 0.1 μM VPC23019 (Tocris, 4195) was supplemented continuously in CDM and CM maintenance medium. Cells were subjected to further analysis for 15 days after the onset of differentiation.

### Fluorescence-activated cell sorting (FACS) analysis

FACS was performed to evaluate the differentiation efficiency of the CMs using a Transcription Factor Buffer Set (BD Biosciences, 562574), as per the manufacturer’s instructions. hPSC-derived CMs were dissociated into single cells using 0.25% Trypsin-EDTA (Gibco, 25200072), which were fixed and permeabilized with TF Fix/Perm Buffer for 30 min at 4 °C, and then washed thrice with Perm/Wash Buffer. Next, anti-cardiac troponin T (cTnT) antibody (Abcam, ab8295) was diluted 1:40 in the Perm/Wash buffer and reacted with cells for 50 min at 4 °C. After washing thrice with Perm/Wash buffer, the cells were resuspended in PBS, and FACS analysis was performed using an Automated High-performance Flow Cytometer (BD Biosciences, FACSverse). FACS data were analyzed using the FlowJo V10 software (TreeStar).

### Reverse transcription–quantitative polymerase chain reaction (qPCR)

Total RNA was extracted using the RNeasy Kit (Qiagen, 74106), and complementary DNA was synthesized using the SuperScript IV First Strand Synthesis System (Invitrogen, LT02241), as per the manufacturer’s instructions. Next, qPCR was performed using a QuantStudio 5 Real-Time PCR Instrument (Life Technologies, A28134). RNA extracted from adult human hearts (Takara, 636532) was used as a positive control. Primers used in this study are listed in Supplementary Table 1.

### Microarray, data acquisition, and analysis

For microarray analysis, total RNA was processed using the Agilent Low Input Quick Amp Labeling Kit (Agilent Technologies, USA), as per the manufacturer’s instructions. Briefly, T7 promoter primers were used to reverse-transcribe RNA to complementary DNA at 40 °C for 2 h. After thermal inactivation, in vitro transcription with cyanine 3-cytidine triphosphate was performed at 40 °C for another 2 h to generate labeled complementary RNA. The labeled complementary RNA was purified (Qiagen), quantified (NanoDrop Technologies), and fragmented before hybridization (at 65 °C for 17 h) to an Agilent Whole Human Genome Microarray 4×44K (EG4112F, Agilent Technologies). Following hybridization, the chip was washed and scanned using an Agilent DNA Microarray Scanner (Agilent Technologies). Data were quantified using the Agilent Feature Extraction software 10.7 (Agilent Technologies). The average fluorescence intensity for each spot was calculated, and the local background was subtracted. All data normalization and selection of genes with fold change were performed using GeneSpringGX 7.3.1 (Agilent Technologies). Normalization for the Agilent One-Color method was performed as follows: data transformation, set measurements of <5.0 to 5.0; per chip, normalized to the 50th percentage. Averages of the normalized ratios were calculated by dividing the averages of the control-normalized and test-normalized signal intensities. Functional annotation of the genes was performed according to the Gene Ontology Consortium (http://www.geneontology.org/index.shtml) using GeneSpringGX 7.3.1.

### Immunofluorescence staining

For immunofluorescence staining, the cells were fixed with 4% paraformaldehyde (PFA) (Biosesang, P2031) for 5 min and permeabilized using 0.1% Triton X-100 (Sigma, T8787) diluted in PBS at 4 °C. After washing with PBS, cells were blocked with 4% bovine serum albumin (BSA; Bovogen, BSAS0.1) and diluted in PBS for 1 h at room temperature. Next, the cells were incubated with primary antibodies (Supplementary Table 2) at 4 °C overnight, followed by incubation with fluorescence-conjugated secondary antibodies for 1 h at room temperature. Nuclei were stained with 4ʹ6-diamidino-2-phenylindole. A fluorescence microscope (1×51, Olympus) and a confocal microscope (FV1000 Live, Olympus) were used for examining the immunofluorescence.

### Electrophysiological characterization using an MEA system

The MEA assay was performed as described previously^22^. Briefly, CMs (4.0 × 10^4^ cells/well) were plated onto MEA plates and incubated. The culture medium was changed every 2–3 days post-seeding. To confirm the activity of CMs, cells cultured for 10 days were transferred to an MEA chamber (37 °C) supplied with a gas mixture (5% CO_2_, 20% O_2_, and 75% N_2_). After achieving a stable field-potential (FP) waveform, the measurements were recorded for 30 min. The spontaneous FPs of the beating CMs were captured using the AxIS software (version 2.4). Data analysis was performed using the Axion Cardiac Data Plotting Tool (Axion BioSystems).

### Measurement of oxygen consumption rate

The oxygen consumption rate (OCR) was measured using a Seahorse XFp analyzer (Agilent, Santa Clara, CA, USA). Before assay, 10,000 cells/well were seeded into a seahorse XFp Cell Culture Miniplate and incubated according to the conditions including control, FBS, S1P, and SEW2871 for 96 h until spontaneous beating was observed. The growth medium was then changed to a Seahorse XF Base Medium containing 2 mM L-glutamine, 5.5 mM D-glucose, and 1 mM sodium pyruvate, according to the composition of α-minimum essential medium. During the assay, cells were treated with 1 μM oligomycin, 1 μM FCCP, and 0.5 μM ronenone/antimycin A. The OCR was normalized according to the protein concentration determined using the BCA assay. The results were then obtained using the manufacturer’s software.

### Guide RNA (gRNA) design

Herein, two pairs of gRNAs were designed using CRISPR RGEN Tools (http://www.rgenome.net/cas-designer/), specifically targeting the Exon2 region of *S1PR1*. The gRNAs were selected according to the criteria recommended by the CRISPR RGEN Tools System. The gRNA pair sequences were as follows: 5ʹ-TTCCCATTTCCCTTTGAGTG-3ʹ, 5ʹ-CCCCAGACAAGAGCAGGTTA-3ʹ, and 5ʹ-CTGTGGCTCTTTCCCTGACT-3ʹ, 5ʹ-AGTTATTGCTCCCGTTGTGG-3ʹ. These gRNA sequences were cloned into a gRNA Cloning Vector (#41824; Addgene).

### S1PR1-knockout (KO) cell-line construction

Plasmid DNA was electroporate using the NEPA21^™^ transfection system, consisting of 1 μg of each of two gRNA pairs, 3 μg of px459 coding Cas9 (Addgene: #62988), 1 μg of pCE-mp53DD coding p53 C-terminal dominant-negative fragment (Addgene: #41856), and 1 μg of pEGFP-N1 into 0.5–1 × 10^6^ cells with the following setting: voltage, 115 V; interval, 50 ms; number, 2; decay rate, 10%; polarity, + for poring pulse; voltage, 20 V; interval, 50 ms; number, 5; decay rate, 40%; polarity, +/− for transfer pulse. For antibiotic selection, 50 μg/ml G418 sulfate (Thermo, Cat. No. 10131027) was treated with 10 μM Y-27632 (Tocris), and it was replaced every day for 4–6 days. The surviving hPSCs without morphological changes were re-seeded as separate single cells in six-well plates. Following this, single colonies were transferred to a 48-well plate. DNA was extracted from some hPSCs for genotyping, whereas the remaining cells were transferred to new six-well plates. qPCR genotyping was performed using the HotStart Taq DNA polymerase (Bioneer, Cat. No. E-2017), corresponding to a 40% KO generation efficiency. The primer sequences are listed in Supplementary Table 1.

### Processing tissue and hematoxylin–eosin (HE) staining

The embryonic and adult mouse heart tissues were prepared as previously described^23,24^. The tissues were fixed with 4% PFA overnight, followed by a serial concentrations of 10%, 20%, and 30% sucrose in PBS overnight. After immersing in 30% sucrose, tissues were embedded in Optimal Cutting Temperature compound (HIO-0051, Sakura, Japan) and sectioned using a Freezing Microtome (Tissue-TEK Polar D, Sakura). After washing with the tap water once to remove any residual compounds, tissues were stained with HE, with hematoxylin staining for 1 min and eosin staining for 1 min after washing once with deionized water. Following this, samples were subjected to dehydration with 80%, 90%, 95%, and 100% ethanol solutions and finally with xylene for 3 min. The completely dehydrated tissue samples were mounted with xylene mounting solution (9999122, Epredia, MI, USA) and carefully covered with glass coverslips. The stained tissue slides were imaged using an inverted microscope (BX53, OLYMPUS, Japan).

### Human phospho-kinase array

To quantify phosphorylation of signaling pathway proteins, the Proteome Profiler Human Phospho-Kinase Array Kit (ARY003, R&D Systems) was used as per the manufacturer’s instructions. Proteins were extracted from untreated hPSC-derived CMs. After lysis at 4 °C using the Lysis Buffer of Proteome Profiler Human Phospho-Kinase Array kit for 30 min, the phospho-kinase array membranes were completely blocked, and 200 μg of the total proteins was incubated on membranes at 4 °C overnight. The detection antibody cocktail was then incubated for 2 h at room temperature. Signals were revealed and images were obtained and quantified using the ImageJ software to determine phospho-protein levels.

### Western blotting

CMs were lysed using the radioimmunoprecipitation assay buffer (Sigma-Aldrich), including 1× phenylmethanesulfonyl fluoride (Sigma-Aldrich), 1 mM protease inhibitor, and 1× phosSTOP (Roche, Indianapolis, IN, USA). After lysis, proteins were quantified using the Pierce BSA assay Kit (Thermo), and 20 μg of protein was loaded into each precast gel (4–20% gradient; Bio-Rad Laboratories, Hercules, CA, USA). During gel electrophoresis, the proteins in the gel were transferred to the membrane. After blocking of membrane with 3% BSA in Tris-buffered saline with Tween 20, the membranes were incubated with the appropriate primary antibodies at 4 °C overnight. The membranes were then incubated with horseradish peroxidase-conjugated secondary antibodies. Finally, the signal was detected using a luminescent image analyzer LAS-3000 (Fuji Photo Film GMBH, Tokyo, Japan).

### Modeling of MI and CM injection

All animal studies were approved by the Animal Care and Use Committee of the Konkuk University of Korea (KUIACUC: KU22019). All applicable ethical regulations for animal testing and research were followed. All Sprague–Dawley rats (age: 7 weeks, male; Orientbio, Korea) received the immunosuppressants cyclosporine A (10 mg/kg) and methylprednisolone (2 mg/kg) daily. Before thoracotomy, rats were anesthetized with 2.5% isoflurane through inhalation and intubated through the trachea using an 18-gauge venous catheter. Simultaneously, they were mechanically ventilated using medical oxygen. After left intercostal thoracotomy, the LAD artery was ligated using a 6-0 propylene suture. MI was induced using an acute MI model based on hypoxia by ligation of the LAD coronary artery with a 6-0 polypropylene suture following left intercostal thoracotomy, without reperfusion^25,26^. Before transplantation, all experimental groups (PBS, control CM, S1P, and SEW2871) were prepared in a solution consisting of 54 μl PBS (with or without cells) and 6 μl Matrigel. For the cell transplantation groups, the suspension was adjusted to contain a total of 3.5 × 10^6^ cells per rat. Subsequently, 10 min after MI induction, the prepared samples were immediately injected into two distinct sites within the border zone of the infarcted myocardium in all groups. As a control, a non-infarcted (non-MI) group without LAD ligation was included. All sampling and analyses were performed 28 days after MI induction and cell transplantation. At this time point, the heart weight of 11-week-old Sprague–Dawley rats was approximately 2.4–2.9 mg/g of body weight, and cardiac transverse sections were ~1–1.3 cm in size^27^.

### Echocardiography

Functional improvement in MI-damaged heart was evaluated using echocardiography. After light isoflurane anesthesia, physiological data on left ventricular (LV) systolic function were recorded using an echocardiography system (GE Vivid 7) 28 days after cell injection. Specifically, the LV end-diastolic dimension (LVEDD) and the LV end-systolic dimension (LVESD) were measured, and fractional shortening (FS) was calculated as follows:

$${\rm{FS}}({\rm{ \% }})=([{\rm{LVEDD}}-{\rm{LVESD}}]/{\rm{LVEDD}})\times 100.$$

### Histological analysis

All rats were euthanized 4 weeks after CM transplantation, and their hearts were collected and fixed with 4% PFA. Fixed samples were embedded in paraffin and 6-μm cross-sections were cut using a HM 340E microtome (ThermoScientific), starting from the apex to the base. Masson’s trichrome (MT) staining was performed to determine the fibrotic areas at MI sites. Paraffin-embedded sections from each group were deparaffinized and fixed in Bouin’s solution at room temperature overnight. Next, fixed sections were stained using Weigert’s iron hematoxylin solution for 10 min at room temperature, followed by treatment with a Biebrich Scarlet-acid Fuchsin solution for 15 min at room temperature. Finally, sections were counterstained with Aniline blue for 5 min. Extensive cleaning was performed after each step. In the MI-damaged heart, the viable myocardium appeared red, and the collagen fibers appeared blue. The percentage of the fibrotic area to the entire LV wall area was quantified using the ImageJ software. Subsequently, to verify the presence of transplanted CMs expressing human nuclear antigen (HNA), immunofluorescence staining was performed after deparaffinization. Primary antibodies against cTnT (Abcam, ab45932) and HNA (Abcam, ab191181) were used. Imaging was performed using a Nikon TE2000-U fluorescence microscope.

### Statistical analysis

All statistical analyses were performed using Microsoft Excel and GraphPad Prism version 10 (GraphPad Software, San Diego, CA, USA). The data are expressed as mean ± SEM. Statistical significance was determined using Student’s *t* test for pairwise comparisons and one-way analysis of variance followed by Holm–Sidak post hoc test for multiple group comparisons, unless otherwise described. Statistical significance is indicated as follows: *P < 0.05, **P < 0.01, and ***P < 0.001.

## Results

### Serum-induced promotion of CM differentiation across hPSC lines

Herein, different cell lines of well-maintained hPSCs, including H9 hESC line and three, respectively, generated hiPSC lines, were differentiated through a modified method to obtain human CMs under the regulation of WNT signal pathway (Supplementary Fig. 1a). After 14 days from the initiation of CM differentiation, the spontaneous beating regions and human CM-specific morphology were observable in all four hPSC lines (Supplementary Fig. 1b and Supplementary Video 1). Flow cytometry results revealed that FBS treatment substantially improved differentiation efficiency, achieving >90% cTnT^+^ cells with low interline variability. Conversely, untreated controls exhibited poor and variable efficiency (46.5–72.2%) (Supplementary Fig. 1c). FBS-induced increased CM differentiation efficiency showed consistency with previous studies. The transcript levels of CM-specific, atrial-specific, and ventricular-specific transcription factors in hPSC-derived CMs showed marked increase in the expression of CM transcription marker genes, including *GATA4*, *NKX2.5*, and *HAND2*, at an earlier stage of differentiation (Supplementary Fig. 1d). Additionally, the expression of atrial (*MYH6* and *MYL7*) and ventricular (*MYL2* and *MYH7*) marker genes was significantly upregulated in FBS-treated CMs (Supplementary Fig. 1d) compared with that in control CMs. Consistently, immunofluorescence results confirmed the upregulated levels of CM marker proteins in FBS-treated cells, including nuclear NK2 homeobox 5 (NKX2.5) and cytoplasmic contractile markers (namely, myosin light chain 2a (MLC2a), ventricular myosin light chain-2 (MLC2v), and cTnT) (Supplementary Fig. 1e). Furthermore, electrophysiological recordings using an MEA showed that FBS-treated CMs presented more synchronized and regular FPs and beat propagation than the controls (Supplementary Fig. 1f,g). These results indicate that FBS-treated CMs formed a more synchronized intercellular network, suggesting that FBS treatment during CM differentiation promoted the differentiation efficiency.

### S1P enhanced hPSC-derived CM differentiation via the S1PR1 signaling axis

The key FBS components that promoted CM differentiation were identified through microarray analysis. The transcriptome of FBS-treated CMs resembled that of an adult human heart tissue as early as day 15, whereas control CMs required >30 days to show a similar profile (Supplementary Fig. 2a). The microarray results revealed the upregulated receptor genes that responded to the soluble factors in the FBS, identifying the key factors promoting CM differentiation efficiency. Although the genes most prominently upregulated in the microarray analysis were cardiac-related transcription factors, including *KLF2*, *STAT3*, *NKX2.5*, and *TBX* family, *S1PR1* was the only gene encoding a membrane-bound receptor, indicating FBS as an upstream activator of S1PR1 signaling (Supplementary Fig. 2b). S1PR1 has been shown to have a critical role in cardiac development. Therefore, S1P, a known serum component and a natural ligand of S1P receptors, was considered a key factor in promoting CM differentiation in this study. Moreover, *S1PR1* was the only gene that showed significant upregulation following FBS treatment, whereas other subtypes of the S1PR family showed no significant differences regardless of FBS treatment (Supplementary Fig. 2c). This pattern showed consistency with the gene expression of each S1PR family member in qPCR results (Supplementary Fig. 2d).

**Fig. 1:**
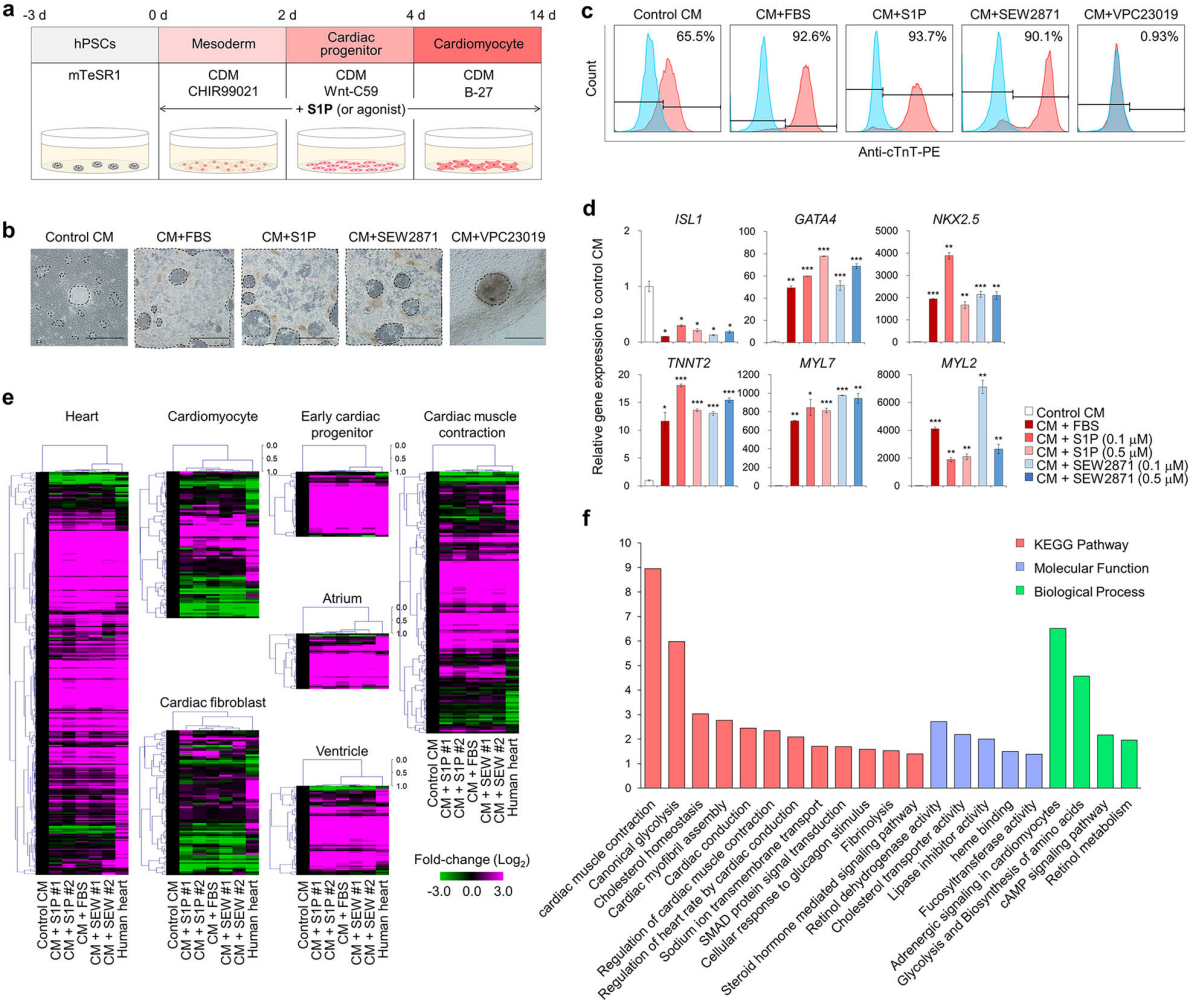
S1P-sphingosine-1-phosphate receptor (S1PR)1 interaction promotes cardiac differentiation from hPSCs. **a** Scheme of CM differentiation protocol with or without S1PR1 signaling pathway activation. **b** Bright-field images showing CM morphologies after 14 days of differentiation from hPSCs treated with FBS, S1P, S1PR1 agonist SEW2871, and S1PR1 antagonist VPC23019. Dashed lines indicate the beating region. Scale bar, 200 μm. **c** Fluorescence-activated cell sorting histograms in control CMs and in CMs treated with FBS, S1P, SEW2871, and VPC23019. **d** Quantitative PCR analysis for the expression of CM-specific marker genes in control CMs and in CMs treated with FBS, S1P, and SEW2871. Data are presented as the mean ± SEM (*n* = 3). **e** Heatmaps of cardiac-specific marker genes in CMs based on RNA sequencing. Human heart RNA was used as a positive control. **f** Gene ontology pathways indicating differentially expressed genes related to cardiac functions and metabolic properties. *P < 0.05, **P < 0.01, ***P < 0.001 using a two-tailed *t* test. CDM, cardiomyocyte differentiation medium; CM, cardiomyocyte; cTnT, cardiac troponin T; FBS, fetal bovine serum; hPSC, human pluripotent stem cell; KEGG, Kyoto Encyclopedia of Genes and Genomes; PE, phycoerythrin; S1P, sphingosine-1-phosphate; SMAD, suppressor of mothers against decapentaplegic homolog.

The functional role of S1PR1 was directly evaluated by subjecting differentiated hPSCs to S1P and its selective agonist, SEW2871, or its antagonist, VPC23019 (Fig. 1a). Notably, expanding spontaneously beating zones were identified in FBS-treated, S1P-treated, and SEW2871-treated CMs, whereas only a few of these zones were observed in the control and VPC23019-treated CMs (Fig. 1b). Consistently, FACS results showed that the S1PR1 activation during CM differentiation after FBS, S1P, or SEW2871 treatment efficiently increased the CM differentiation (90.1–93.7%) compared with the S1PR1-specific antagonist VPC23019 (0.93%) (Fig. 1c). Consistently, the expression of mature CM marker genes, including *NKX2.5*, *GATA4*, *MYL7*, *MYL2*, and *TNNT*, was markedly higher in the FBS, S1P, and SEW2871 groups than in the control CM group, whereas the expression of the early cardiac lineage marker *ISL1* was relatively higher in the control group (Fig. 1d). Improvement in CM differentiation of S1PR1-activated cells was further validated through an messenger RNA-sequencing-based comparative analysis. Compared with control CMs, RNA profiles of CMs treated with FBS, S1P, or SEW2871 highly resembled those of the human heart samples in gene sets related to the heart, CM, cardiac fibroblasts, early cardiac progenitors, atria, ventricles, and cardiac muscle contractions (Fig. 1e). Additionally, cardiac muscle contraction function-related and cardiac conduction-related genes showed significant enrichment, highlighting metabolic changes (Fig. 1f). Overall, these data indicate that S1P-treated or SEW2871-treated CMs robustly enhanced the differentiation of hPSCs into CMs.

### Morphological and functional maturation of hPSC-derived CM via S1P–S1PR1 signaling

**Fig. 2:**
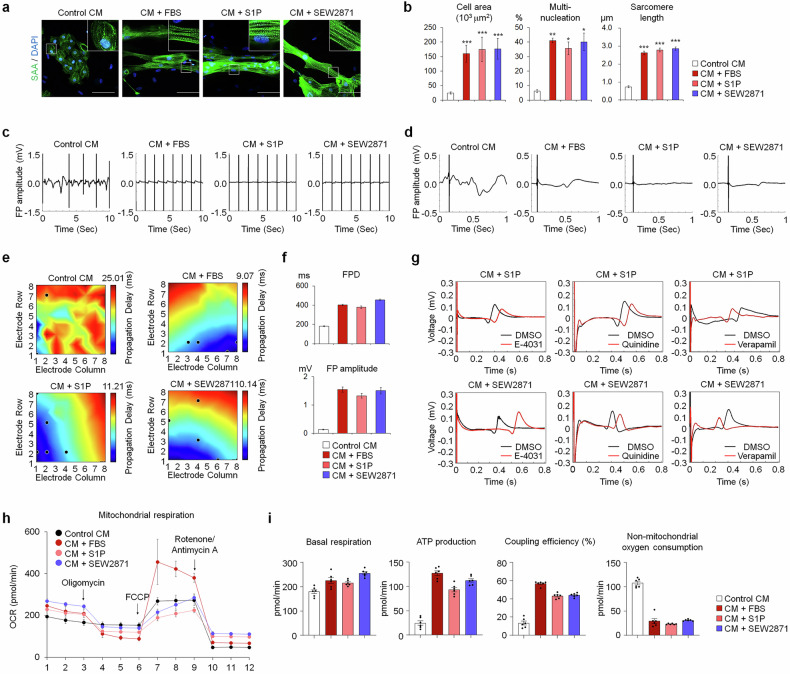
S1P-sphingosine-1-phosphate receptor 1 interaction during CM differentiation enhances maturity and functions. **a** Immunofluorescent staining for SAA and average cell area of single CMs. Scale bar, 50 μm. **b** Cell area, quantitative percentage of multinucleated CMs in the field, and sarcomere length. Data are presented as mean ± SEM (*n* = 3). **c**, **d** The traces of the multielectrode array recording from control CMs and CMs treated with FBS, S1P, and SEW2871. **e** Representative images of beat propagation. **f** FPD (upper panel) and FP amplitude (lower panel) obtained from the control CMs and CMs treated with FBS, S1P, and SEW2871. **g** Changes in representative raw traces following treatment with cardiac ion channel inhibitors in CMs treated with S1P or SEW2871. E-4031: potassium channel, quinidine: sodium channel, and verapamil: calcium channel. **h** Comprehensive oxygen consumption rates in control, FBS-treated, S1P-treated, or SEW2871-treated CMs. **i** Graphs of the basal respiration, ATP production, coupling efficiency, and non-mitochondrial oxygen consumption. CM, cardiomyocyte; DAPI, 4ʹ,6-diamidino-2-phenylindole; DMSO, dimethyl sulfoxide; FBS, fetal bovine serum; FP, field potential; FPD, field potential duration; OCR, oxygen consumption rate; S1P, sphingosine-1-phosphate; SAA, sarcomeric alpha-actinin. All experiments were performed thrice; similar results were noted each time, and representative experiments are shown. *P < 0.05, **P < 0.01, ***P < 0.001 using a two-tailed *t* test.

CM functionality is characterized by an enlarged size and organized structure. Therefore, CM’s functionality was quantified based on the geometry of the immunostained CMs, including the cell area, multinucleation ratio, and sarcomere length. Notably, the immunofluorescent staining images revealed that FBS-treated, S1P-treated, or SEW2871-treated CMs showed robust crosslinking networks of sarcomeric alpha-actinin, whereas the control CMs exhibited a lower expression and less interconnected networks of sarcomeric alpha-actinin (Fig. 2a). Moreover, the overall cell area, multinucleation ratio, and sarcomere length were significantly higher in FBS-treated, S1P-treated, or SEW2871-treated CMs than in the control group (Fig. 2b). Next, the MEA assay results compared the electrophysiological functionality of each CM group. Raw traces of the MEA recordings indicated that FBS-treated, S1P-treated, or SEW2871-treated CMs showed a more regular FP pattern than control CMs (Fig. 2c,d). Reportedly, the normal range for QTc is 350–450 ms for adult men and 360–460 ms for adult women^28^. Herein, the FP duration (FPD) of S1PR1-activated CMs ranged from 378 ms to 455 ms, showing values more similar to the FPD of human heart CMs. By contrast, the FPD of the control group was 179 ms, indicating that S1PR1-activated CMs closely resembled mature human hearts. It was observed that S1PR1-activated CMs exhibited significantly larger FP amplitudes than the control group. Moreover, the cardiac conduction plot indicated regular and synchronous beatings observed in S1PR1-activated CMs, whereas control CMs showed spatiotemporally irregular patterns of beating (Fig. 2e,f). Along with their biological properties, the electrophysiological functions of S1P-treated or SEW2871-treated CMs were found to be disrupted by selective ion channel inhibitors, including E-4031 (10 nM), quinidine (300 nM), and verapamil (30 nM). These findings show consistency with previous studies^29–32^; notably, E-4031 lengthened the duration, quinidine slightly decreased the amplitude, and verapamil shortened the duration of electrophysiological parameters compared with the dimethyl sulfoxide-treated group (Fig. 2g). Additionally, metabolic functionality of CMs was evaluated by measuring mitochondrial respiration. OCR analysis revealed that S1P-treated CMs exhibited significantly increased basal respiration and ATP production compared with control CMs, indicating enhanced mitochondrial activity following differentiation (Fig. 2h). Coupling efficiency, reflecting the proportion of oxygen consumption utilized for ATP production, was also markedly elevated in FBS-treated, S1P-treated, or SEW2871-treated CMs, suggesting more efficient oxidative phosphorylation (Fig. 2i). By contrast, control CMs showed higher non-mitochondrial oxygen consumption, consistent with reduced mitochondrial engagement and a less mature metabolic phenotype (Fig. 2i). Collectively, these metabolic profiling results confirm that the activation of S1PR1 signaling enhances mitochondrial function and energy metabolism, further substantiating the functional maturation of CMs.

Interestingly, the lactate enrichment strategy, which reportedly improves CM maturation by increasing purity and functionality^33–35^, did not cause any substantial difference in cell morphology or expansion of the beating area compared with that of S1PR1-activated CMs (Supplementary Fig. 3a and Supplementary Video 2). Similarly, lactate enrichment significantly increased the population of cTnT-positive CMs and markers for mature CM or various ion channels compared with that in control CMs, but not in S1P-treated or SEW2871-treated CMs (Supplementary Fig. 3b–d). Collectively, these results indicate that the activation of S1P–S1PR1 signaling pathway robustly stimulated CM differentiation of hPSCs with high efficiency and functional maturity. Moreover, S1PR1 activation triggered the maturation of human CMs, which is comparable to the in vivo level of cardiac tissue in terms of functionality.

### CRISPR–Cas9-mediated S1PR1 disruption inhibited CM differentiation and functional integration

**Fig. 3:**
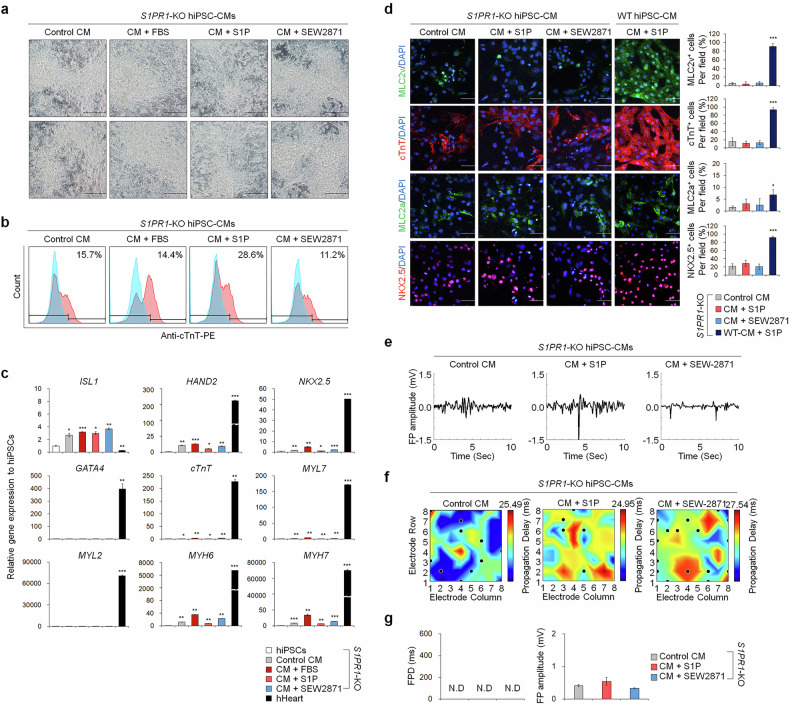
Defective CM differentiation of S1PR1-KO hiPSCs. **a** Bright-field images of the morphologies of S1PR1-KO hiPSC CMs at 14 days after initial differentiation. Scale bar, 200 μm. **b** Fluorescence-activated cell sorting histograms showing cTnT-positive cell population in S1PR1-KO hiPSC-derived CMs treated with or without FBS, S1P, and SEW2871. **c** Quantitative PCR data showing CM marker gene expression in S1PR1-KO hiPSC CMs. Data are presented as mean ± SEM (*n* = 3). **d** Immunofluorescent staining for CM-specific marker proteins and comparison of quantitative percentage between groups. Scale bar, 50 μm. Data are presented as mean ± SD (*n* = 3 biological samples). **e** The trace of the multielectrode array recording from control CMs and CMs treated with S1P, and SEW-2871 derived from S1PR1-KO hiPSCs. **f** Representative images exhibiting beat propagation of each group. **g** Quantitative analysis of FPD and FP amplitude in S1PR1-KO hiPSC-derived CMs. Data are presented as mean ± standard error of the mean. All experiments were performed thrice; similar results were observed each time, and representative experiments are shown. *P < 0.05, **P < 0.01, ***P < 0.001 using a two-tailed *t* test. CM, cardiomyocyte; cTnT, cardiac troponin T; DAPI, 4ʹ,6-diamidino-2-phenylindole; FBS, fetal bovine serum; FP, field potential; FPD, field potential duration; hiPSC, human-induced pluripotent stem cell; KO, knockout; MLC2a, myosin light chain 2a; MLC2v, ventricular myosin light chain-2; N.D., not detected; NKX2.5, NK2 homeobox 5; PE, phycoerythrin; S1P, sphingosine-1-phosphate; S1PR, sphingosine-1-phosphate receptor.

The role of S1PR1 in CM differentiation was verified by comparative analysis of three *S1PR1*-KO hiPSC clones generated using the CRISPR–Cas9 gene editing system. The exon 2 of *S1PR1* was deleted utilizing a pair of S1PR1-targeting single gRNAs (Supplementary Fig. 4a), and the successful KO of *S1PR1* was confirmed in *S1PR1*-KO hiPSC lines (Supplementary Fig. 4b). Additionally, qPCR data showed the expression of *S1PR1* to be completely diminished in *S1PR1*-KO hiPSC lines (Supplementary Fig. 4c). Immunofluorescence staining revealed that *S1PR1*-KO hiPSC lines maintained the features of hPSCs without any abnormalities, even after gene editing (Supplementary Fig. 4d,e). The pluripotency characteristics of *S1PR1*-KO hiPSCs were identified and these cells were differentiated into CMs with or without treatment with FBS, S1P, or SEW2871. Fourteen days after the initial CM induction, only a few beating regions were observed in all conditions of *S1PR1*-KO hiPSC-CMs, which were different from previous results (Fig. 3a). Despite the treatment with S1PR1 activators, *S1PR1*-KO hiPSC-derived CMs revealed a markedly diminished population of cTnT-positive CMs (19.4–43.3%) (Fig. 3b), along with the decreasing expression of CM marker genes (Fig. 3c). Additionally, the immunofluorescence staining results showed that *S1PR1*-KO hiPSC-CMs had a much lower number of cells expressing CM-specific marker proteins, including MLC2a, MLC2v, cTnT, and NKX2.5, than wild-type (WT) hiPSC-CMs differentiated by S1PR1 activation (Fig. 3d). Similarly, the electrophysiological analysis showed irregular FP patterns (Fig. 3e) and propagation (Fig. 3f). Quantitative data further showed low functionality of CMs differentiated from *S1PR1*-KO hiPSCs (Fig. 3g). These *S1PR1*-KO hiPSC-derived CM data collectively indicate that the S1P–S1PR1 signaling pathway has a critical role in improving CM differentiation efficiency and quality.

### P38–mitogen-activated protein kinase (MAPK) signaling functions as a downstream effector of S1P–S1PR1-mediated CM differentiation

**Fig. 4:**
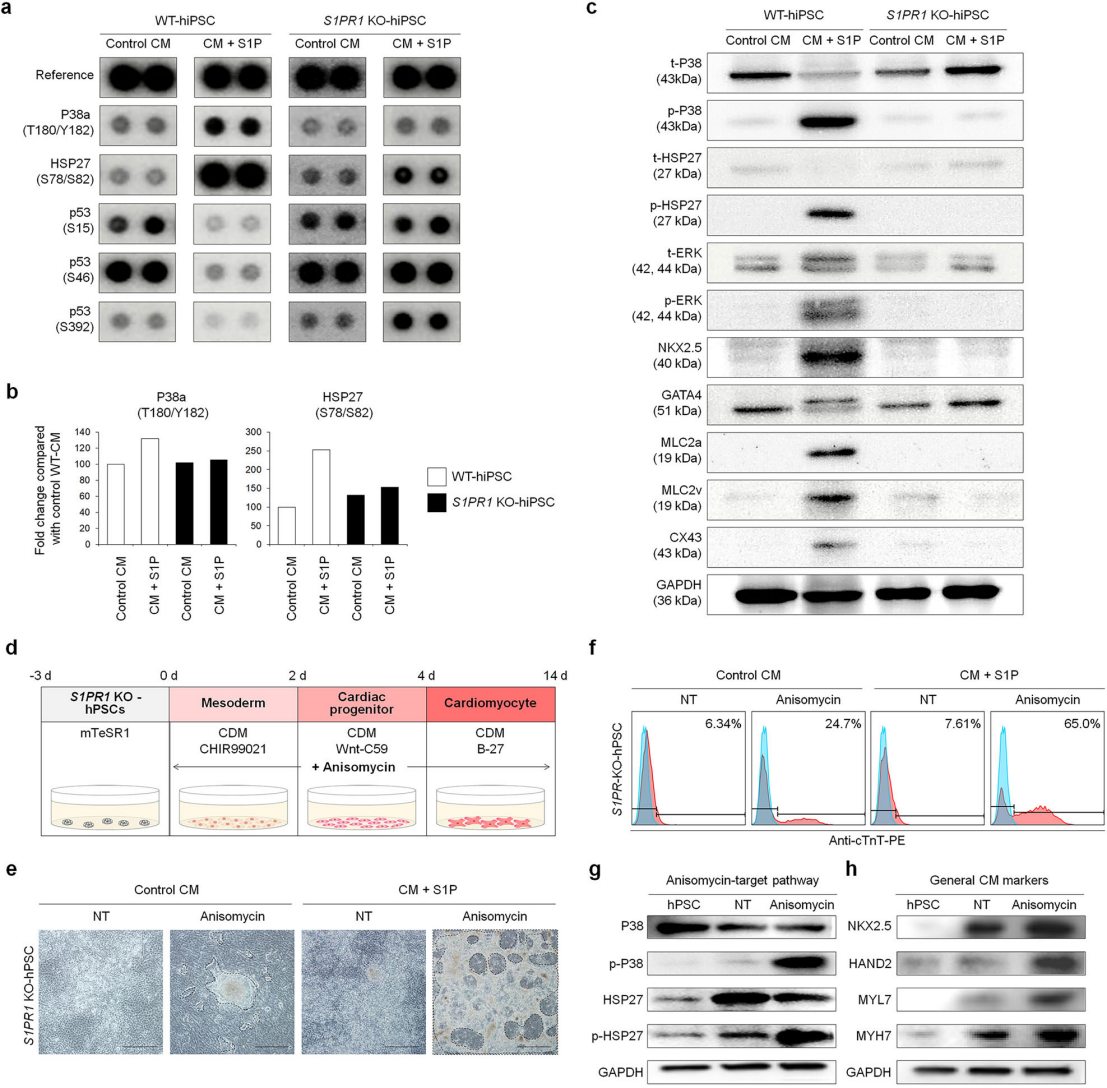
Activated p38 pathway improves CM differentiation of S1PR1-KO hiPSCs. **a** Phospho-kinase array exhibiting phosphorylation levels of protein-related signaling pathways in control and S1PR1-activated CMs differentiated from control or S1PR1-KO hiPSCs. **b** Comparison of phospho-proteins in p38 and HSP27 under the S1PR1-activated conditions. **c** Western blot analysis of representative markers for cardiac differentiation through the p38–HSP27 signaling pathway. **d** Scheme of methods for CM differentiation from S1PR1-KO hiPSCs with p38 activator anisomycin. **e** Representative morphologies of S1PR1-KO hiPSC-derived CMs differentiated with anisomycin. Scale bar, 200 μm. **f** Fluorescence-activated cell sorting analysis for comparison of differentiation yield in the presence or absence of anisomycin. **g**, **h** Western blot analysis showing protein expression of S1PR1-KO iPSC-derived CMs with or without the treatment of anisomycin. CM, cardiomyocyte; CX43, connexin 43; ERK, extracellular signal-regulated kinase; GAPDH, glyceraldehyde-3-phosphate dehydrogenase; GATA4, GATA binding protein 4; hiPSC, human-induced pluripotent stem cell; hPSC, human pluripotent stem cell; HSP, heat-shock protein; iPSC, induced pluripotent stem cell; KO, knockout; MLC2a, myosin light chain 2a; MLC2v, ventricular myosin light chain-2; MYH7, myosin heavy chain 7; MYL7, myosin light chain 7; NKX2.5, NK2 homeobox 5; NT, not detected; S1P, sphingosine-1-phosphate; S1PR, sphingosine-1-phosphate receptor; WT, wild-type.

A phospho-kinase array analysis was performed in WT and *S1PR1*-KO hiPSC-CMs to determine the key downstream signaling pathways activated by the S1P–S1PR1 interaction. Notably, P38 and heat-shock protein (HSP)27 were upregulated only in WT hiPSC-CMs with S1P, but not in *S1PR1*-KO hiPSC-CMs under the same conditions (Fig. 4a,b). Increased phosphorylation of P38, extracellular signal-regulated kinase, and HSP27, which trigger mesoderm and cardiac transcription factors for cardiac development^34,36,37^, was confirmed via western blotting (Fig. 4c). Furthermore, quantitative data showed low functionality of CMs differentiated from *S1PR1*-KO hiPSCs (Fig. 3e–g). The S1P–S1PR1 signaling pathway has a critical role in improving CM differentiation efficiency and quality; hence, the effect of anisomycin on the recovery of disrupted differentiation ability of S1PR1-KO hiPSCs was evaluated, via activated p38–MAPK axis (Fig. 4d). Herein, CM differentiated from S1PR1-KO iPSCs was successfully rescued with anisomycin treatment compared with that in the untreated groups (Fig. 4e and Supplementary Video 3). Notably, when S1PR1-KO iPSCs were treated with anisomycin, the differentiation efficiency increased to 24.7% (Fig. 4f). Furthermore, co-treatment with S1P and anisomycin markedly enhanced the efficiency to 56.0%, suggesting that S1P may synergistically promote CM differentiation (Fig. 4f). Consistently, anisomycin-treated S1PR1-KO CMs exhibited upregulated protein levels, including general CM markers NKX2.5, HAND2, myosin light chain 7, and myosin heavy chain 7, together with an activated p38 pathway, compared with that in S1PR1-KO CMs without anisomycin treatment (Fig. 4g,h). Altogether, these results show that the P38–MAPK signal pathway is a key signaling pathway downstream of the S1P–S1PR1 interaction for enhanced CM differentiation.

### Therapeutic efficacy of S1P-treated and SEW2871-treated CMs in post-MI cardiac repair

**Fig. 5:**
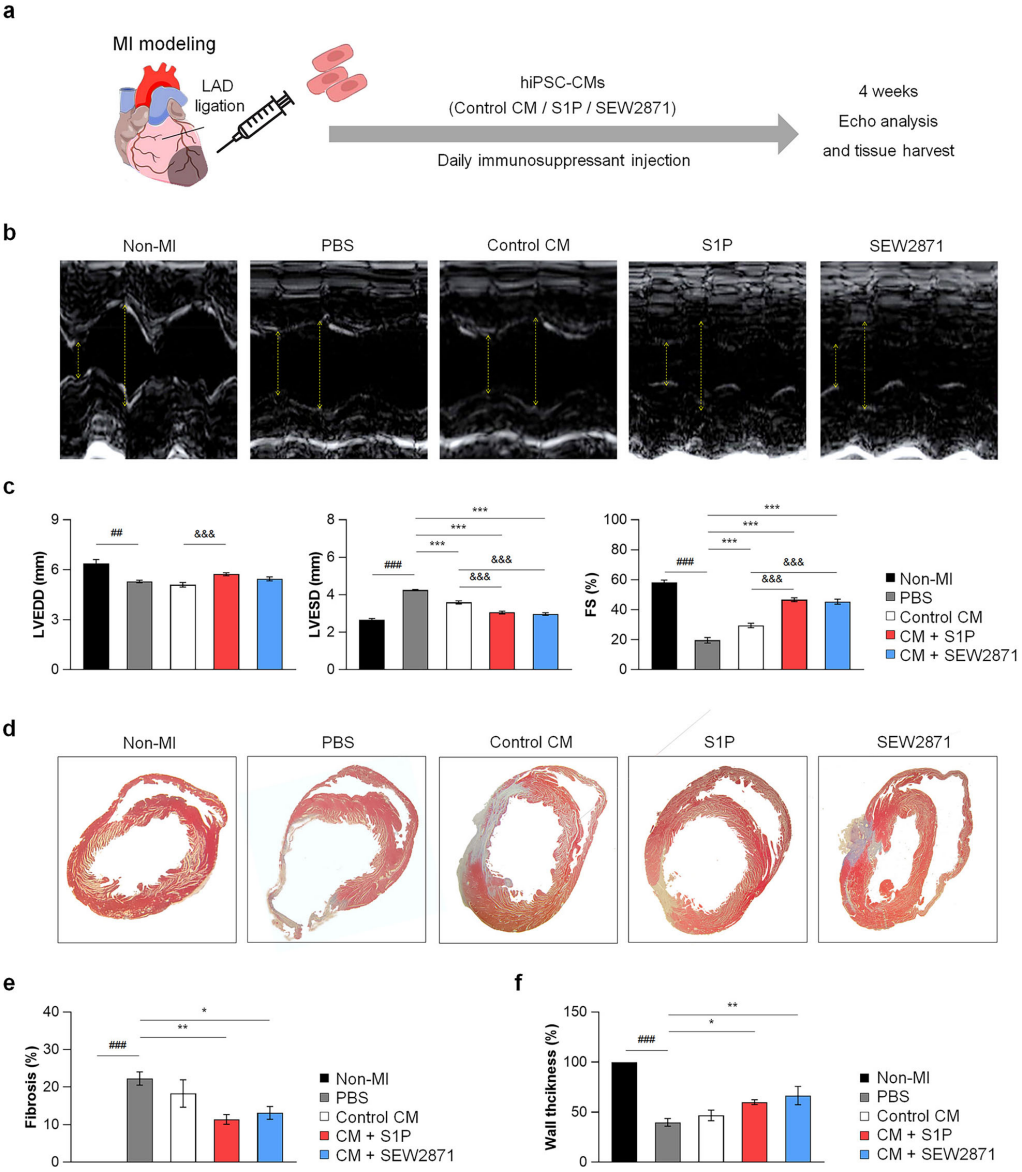
Verification of the therapeutic effect of injected CMs in the MI model. **a** A schematic diagram of the strategy to verify the therapeutic effects of the PBS and CM groups (control CM, S1P, and SEW2871) after MI induction. **b** Echocardiography measurement 28 days after MI induction and injection of CMs (non-MI, PBS, control CM, S1P, and SEW2871; *n* = 5). Echocardiography images of left ventricle between groups. Yellow dotted lines indicate LVESD and LVEDD. **c** Comparison of LVEDD, LVESD, and FS between groups. **d** Masson’s trichrome (MT) staining of samples 28 days after MI induction and injection of CMs (non-MI: *n* = 3; PBS: *n* = 8; control CM, S1P, and SEW2871: *n* = 3–4). Comparison of fibrosis (part **e**) and wall thickening in fibrosis area (part **f**) between groups. All quantitative analyses were performed with *n* ≥ 3 biological replicates and data are presented as mean ± standard error of the mean (^##^*P* < 0.01 and ^###^*P* < 0.001, comparison with non-MI; ^*^P < 0.05, ^**^P < 0.01, and ^***^P < 0.001, comparison with PBS; ^&&&^*P* < 0.001, comparison with control CM; ns indicates no significance). CM, cardiomyocyte; FS, fractional shortening; hiPSC, human-induced pluripotent stem cell; LAD, left anterior descending; LVEDD, left ventricular end-diastolic dimension; LVESD, left ventricular end-systolic dimension; MI, myocardial infarction; S1P, sphingosine-1-phosphate.

To evaluate the therapeutic potential of S1PR1 activator-treated CMs, control CMs, S1P-treated CMs, SEW2871-treated CMs, and only PBS (as a negative control) were injected into the MI mouse model generated by LAD artery ligation, which induced ischemic injury in the LV^38^ (Supplementary Fig. 5). To minimize immune rejection of the transplanted human cells, immunosuppressant injections were administered daily (Fig. 5a). Cardiac function was assessed via echocardiography, focussing on LVESD, LVEDD, and FS measurements^39^ (Fig. 5b,c). Compared with the non-MI group, the PBS group exhibited impaired cardiac function following MI induction, as evidenced by altered LV dimensions and a significant reduction in FS (Fig. 5b,c). Compared with the PBS group, all CM-transplanted groups showed significant improvements in cardiac function, including a decrease in LVESD and an increase in FS. Among the CM-transplanted groups, the S1P-treated and SEW2871-treated CM groups demonstrated greater improvements in LVESD and FS than the control CM group. A significant increase in LVEDD was observed only in the S1P-treated CM group compared with the control CM group (Fig. 5c).

MT staining results confirmed the histological results. In MI, CM necrosis within the LV myocardial layer leads to LV wall thinning and extensive fibrosis^26,40^. MT staining revealed these tissue-specific environmental changes. All MI-induced groups exhibited thinner LV wall-stained fibrotic lesions (Fig. 5d,e), with the PBS and control CM groups showing severe fibrosis and wall thinning compared with that in the normal heart (Fig. 5d–f). Notably, transplantation of S1P-treated and SEW2871-treated CMs significantly reduced the fibrotic area and preserved LV wall thickness. cTnT staining of the infarcted myocardium revealed extensive CMs necrosis; however, the S1P-treated and SEW2871-treated groups exhibited a markedly higher CM presence than the PBS and control CM groups (Supplementary Fig. 6). To evaluate the engraftment efficiency of transplanted hPSC-derived CMs within the myocardium, co-immunostaining for cTnT and HNA was performed, which revealed that the proportion of HNA⁺ cells within the cTnT⁺ myocardium region was significantly higher in the S1P-treated and SEW2871-treated CM groups than in the control CM group (Supplementary Fig. 6). Collectively, these findings suggest that S1PR1 activator-treated CMs exhibit enhanced engraftment within the myocardium, thus contributing to superior functional and structural recovery in the post-MI heart compared with untreated or control CM groups.

## Discussion

With advances in stem cell differentiation and development, protocols for generating PSC-derived CMs have been widely investigated to exploit their potential applications in cardiac disease modeling, evaluating drug toxicity, and regenerative therapy. However, challenges, such as fetal-like level of maturity and functionality of hPSC-derived CMs, often limit their application. In this study, S1PR1 signaling was identified as a key modulator that enhanced both the differentiation efficiency and functional maturation of hPSC-derived CMs under chemically defined conditions.

FBS treatment has been extensively utilized to enhance the differentiation efficiency of CMs and other cell types. However, various challenges, including the compositional uncertainty and xenobiotic properties beyond the economical and ethical problems, make FBS unsuitable for defined culture methods and clinical application^41^. Nevertheless, the results of this study confirmed the enhanced CM differentiation following FBS treatment, identifying the S1P–S1PR1 interaction as a key factor in the promotion of differentiation of hPSC-derived CMs. Notably, activating S1PR1 signaling pathway by treatment with either S1P or its agonist, SEW2871, significantly enhanced hPSC-derived CMs’ differentiation efficiency and functional maturity compared with that in the FBS-treated group and the adult heart tissue. S1PR1-activated CMs exhibited a higher population of cTnT-positive cells with upregulated CM marker gene expression, and they also showed regular and synchronous electrophysiological properties originating from mature CM morphology. Reportedly, S1PR1 predominantly expresses in CMs, whereas cardiac fibroblasts express mainly S1PR3 and vascular smooth muscle cells express S1PR2 and S1PR3 (refs. ^42–44^). Considering that the practical application of hPSC-derived CMs in cardiac therapy, drug screening, and disease modeling is mostly limited by immature phenotypes, the proposed method showed a high yield of more mature functional hPSC-derived CMs via the activated S1P–S1PR1 interaction, which sheds light on future cardiac cell-based technologies. Interestingly, the microarray data showed that S1P-treated or SEW2871-treated CMs highly resembled the human heart sample regarding gene sets related to the heart, CM, cardiac fibroblasts, early cardiac progenitors, atrium, ventricle, and cardiac muscle contraction, further supporting the liability of mature PSC-derived CMs.

In the present study, the central signaling pathway promoting CM signaling was identified utilizing S1PR1-KO hiPSCs edited using the CRISPR–Cas9 system. S1PR1-KO hiPSCs were differentiated into CMs under FBS, S1P, or SEW2871 conditions, and no significant upregulated differentiation efficiency and maturity were observed in either condition. Consistently, many studies have shown the defects in vascular development and embryonic lethality both in global S1PR1-KO mice in and Sphk1/2-KO mice^45–48^. For instance, Clay et al.^49^ reported the defects in myocardial development and embryonic lethality of CM-specific S1PR1-KO mice using Mlc2a-Cre conditional mutants. S1P has critical roles in cardiac physiology, including cardiac protection and contractility^18,46,49^, and the enhanced differentiation of CM treated with S1P activators and its inhibition in the S1PR1-KO hiPSCs showed that S1P contributed both to the maintenance of cardiac physiology, such as cardiac protection and contractility, and to the lineage specification during the early heart development. Consistently, anisomycin treatment, which reportedly activates the p38–MAPK pathway downstream of S1PR1, was shown to successfully rescue S1PR1-KO hiPSC differentiation into CM. The p38–MAPK axis has been shown to have important roles in placental angiogenesis, vascular remodeling, and trophoblast and skeletal muscle development, along with the cardiogenic role of p38 both in vivo and in vitro^45,50,51^. For instance, p38 inhibition can decrease the cardiac transcription factors, including myocyte enhancer factor 2C, cardiac structural proteins, atrial natriuretic factor, and myocardin^45,50,52^. Furthermore, p38 can regulate sarcomere assembly by phosphorylating MLC2v and accumulating α-actinin^53^. Aouadi et al.^52^ reported that p38α inhibition or gene deletion could effectively block cardiomyogenesis. Altogether, the enhanced CM differentiation efficiency and maturity via activated S1PR1–P38–MAPK signaling pathway may further validate the significant roles of S1P ligands both in CM differentiation and in heart development.

In the MI model, S1PR1 activator-treated CM groups exhibited superior therapeutic efficacy compared with control CM. The transplantation of S1P-treated and SEW2871-treated CMs led to significantly improved FS, along with reduced myocardial fibrosis and attenuation of LV wall thinning, indicating enhanced recovery of cardiac structure and function (Fig. 5). To explore the mechanism underlying these therapeutic benefits, the engraftment capacity of transplanted human CMs was assessed using cTnT and HNA co-immunostaining. S1PR1 activator-matured CMs showed a significantly higher engraftment rate within the myocardium than control CM (Supplementary Fig. 6), consistent with the findings of previous reports demonstrating superior engraftment of maturation-enhanced CMs (differentiation 56 days) compared with immature CMs (differentiation 28 days)^54^, which may contribute to myocardial wall preservation and functional recovery. Despite these advantages, the MI model used in this study relied on pharmacological immunosuppression rather than a fully immunodeficient or humanized model, thereby presenting a limitation. Considering the potential for immune-mediated rejection and rapid loss of xenotransplanted cells, the post-transplantation observation period was limited to 4 weeks^55,56^. Long-term monitoring of cell engraftment and functional persistence is essential for the clinical translation of hPSC-derived CMs as therapeutic agents. In this context, recent studies have reported strategies for enhancing CM maturation and early engraftment by culturing cells within or by co-injecting them with specialized materials or hydrogels^57,58^. Combining such biomaterial-based approaches with S1PR1 activator-mediated CM maturation may present a promising strategy to accelerate CM maturation and improve engraftment efficiency before and after transplantation.

In summary, this study proposed a novel xenogenic-free chemically defined method to generate hPSC-derived CMs with high purity and maturity by treatment with S1PR1 activators. The generated CMs demonstrated a more similar gene expression pattern to the heart tissue of an adult human and exhibited a more regular and synchronized cellular behavior than control CMs. Previous CM differentiation methods are limited by premature phenotypes with low purity and functionality, and the S1PR1 activator-treated CMs generated through the proposed method address these limitations and may facilitate advanced and cost-effective applications in drug discovery and regenerative medicine research. Furthermore, the importance of S1PR1 in heart development and its association with heart diseases, such as LV non-compaction cardiomyopathy, is assumed to contribute to the identification of complex heart development and disease development mechanisms.

## Supplementary information

Supplementary Information

Supplementary Video 1

Supplementary Video 2

Supplementary Video 3

## Data Availability

The data that support the findings of this study are available from the corresponding author upon reasonable request.
