# Supplementary material for: Sphingosine-1-phosphate receptor 1 signaling stimulates human pluripotent stem cell-derived cardiomyocyte differentiation and maturation
Source: Exp Mol Med. 2026 Jul 2;58(7):2223–34. doi: 10.1038/s12276-026-01767-3 (PMC13434232; doi:10.1038/s12276-026-01767-3)
Supplement: Supplementary file 1 [file 12276_2026_1767_MOESM1_ESM.pdf]

Supplementary Information

Sphingosine-1-phosphate receptor 1 signaling stimulates human pluripotent stem cell-derived cardiomyocyte differentiation and maturation

Won Dong Yu[1,2,†], Seul-Gi Lee[3,†], Kwang Bo Jung[1], Sung-Ae Hyun[4], Young-Dae Kim[1], Ye Seul Son[1], Hana Lee[1], Hyun-Soo Cho[1,2], Dae-Soo Kim[1,2], Kyung Jin Lee[5], Jongman Yoo[5,6], C-Yoon Kim[3], Mi-Ok Lee[1,2,*], Ohman Kwon[1,2,*], Mi-Young Son[1,2,7,*]

[1]Korea Research Institute of Bioscience and Biotechnology (KRIBB), Daejeon, 34141, Republic of Korea[2]KRIBB School of Bioscience, Korea University of Science and Technology (UST), Daejeon, 34113, Republic of Korea; [3]College of Veterinary Medicine, Konkuk University, Seoul, 05029, Republic of Korea; [4]Research Group for Safety Pharmacology, Korea Institute of Toxicology, KRICT, Daejeon, 34114, Republic of Korea; [5]R&D Institute, ORGANOIDSCIENCES, Seongnam, 13493, Republic of Korea; [6]CHA Organoid Research Center, CHA University, Seongnam, 13488, Republic of Korea; [7]School of Medicine, Sungkyunkwan University, Suwon, 16419, Republic of Korea

[†]These authors contributed equally to this work.

[*]**Corresponding authors:** Mi-Ok Lee; molee@kribb.re.kr; Ohman Kwon; omkwon@kribb.re.kr; Mi-Young Son; myson@kribb.re.kr

Supplementary Figures

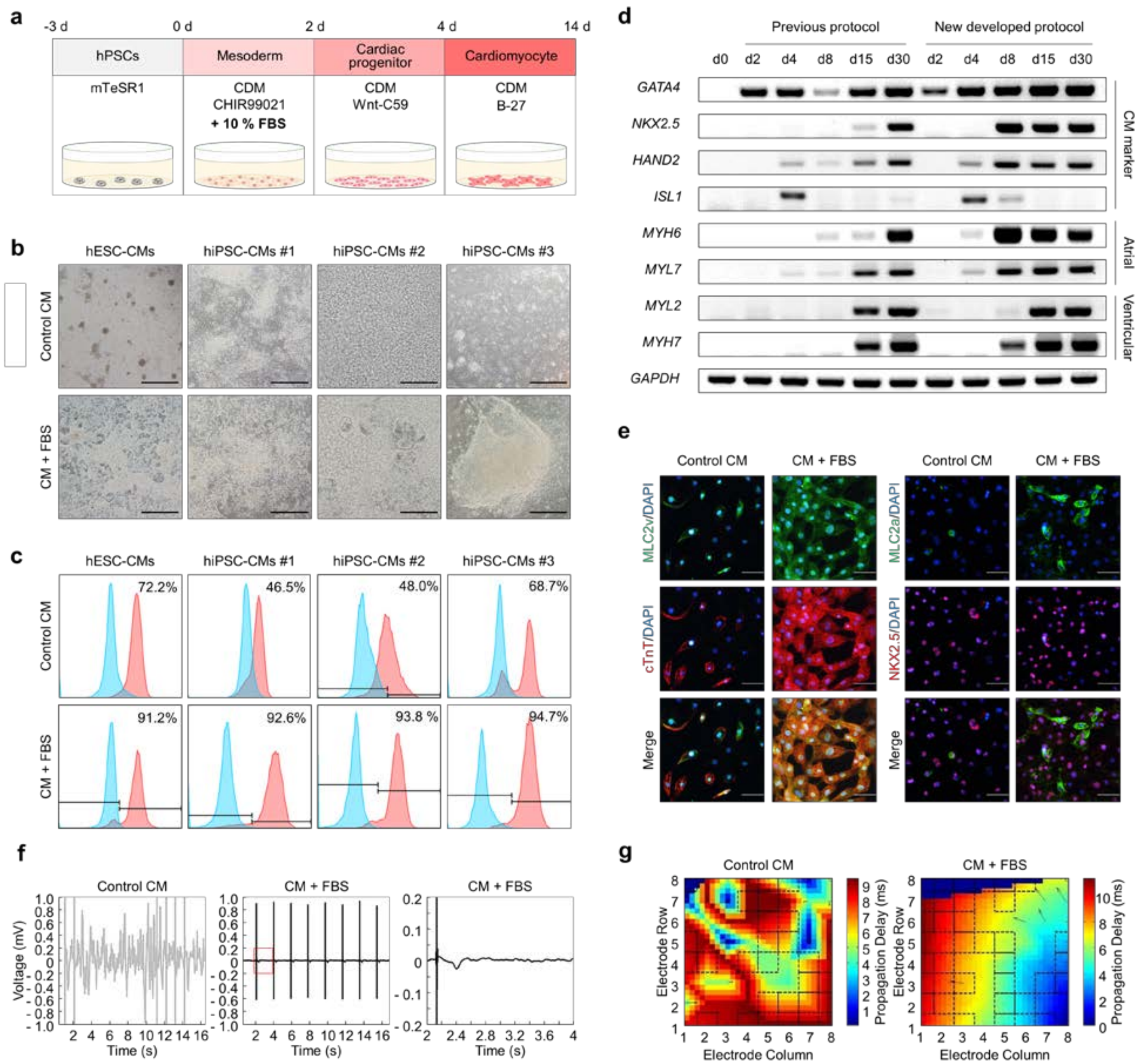

Supplementary Fig. 1. FBS enhances the differentiation efficiency of hPSCs into CMs. a Scheme of the CM differentiation with or without FBS treatment. **b** Bright-field images showing the morphology of hPSC-derived CMs differentiated according to the scheme. Scale bar, 200 μm. **c** Flow cytometry histogram of cTnT-positive CM populations differentiated from hPSCs. **d**

Relative gene expression of CM-specific markers in CMs differentiated with or without FBS. **e** Immunofluorescence staining of CMs for CM-specific marker proteins. Scale bar, 50 μm. **f** Raw traces of MEA recordings from control and FBS-treated CMs. **g** Representative images of the spontaneous beating propagation in control and FBS-treated CMs. hPSC, human pluripotent stem cell; hESC, human embryonic stem cell; hiPSC, human induced pluripotent stem cell; CM, cardiomyocyte; DAPI, 4',6-diamidino-2-phenylindole; MLC2v, ventricular myosin light chain-2; FBS, fetal bovine serum; cTnT, cardiac troponin T; CDM, cardiomyocyte differentiation medium.

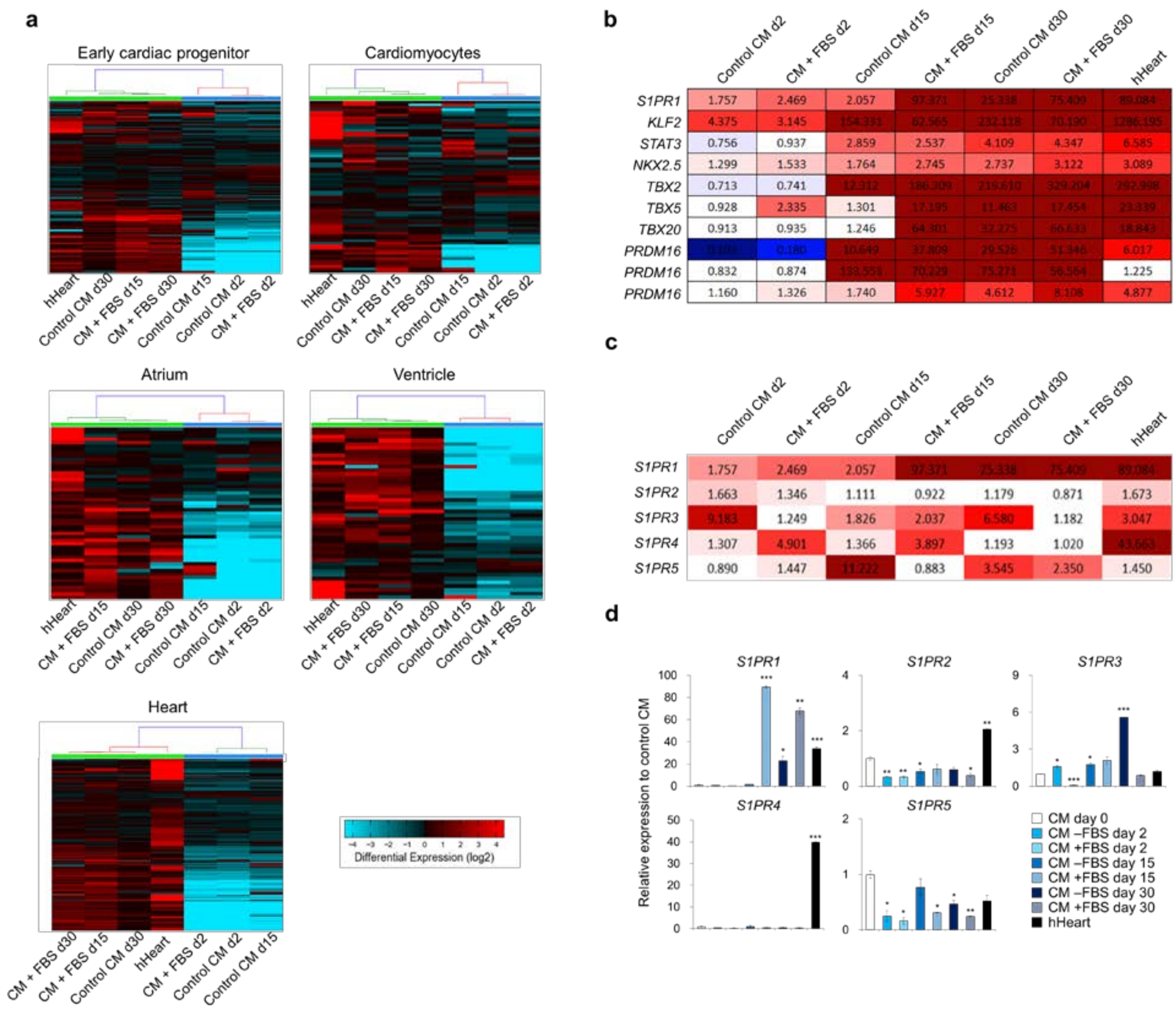

Supplementary Fig. 2. Microarray data analysis of control and FBS-treated CMs. a Heatmap of genes related to the early cardiac progenitor, CM, heart, atrium, and ventricle markers based on microarray results. **b** Representative upregulated genes in the presence or absence of FBS during CM differentiation. **c** Comparative gene expression panel of sphingosine-1-phosphate receptor (S1PR) isoforms in the presence or absence of FBS during CM differentiation based on the microarray analysis. **d** Quantitative polymerase chain reaction analysis of the gene expression of S1PR isoforms. Data are presented as the mean ± SEM (n = 3). * $p < 0.05$, ** $p < 0.01$, *** $p < 0.001$ using a two-tailed *t*-test. FBS, fetal bovine serum; CM, cardiomyocyte.

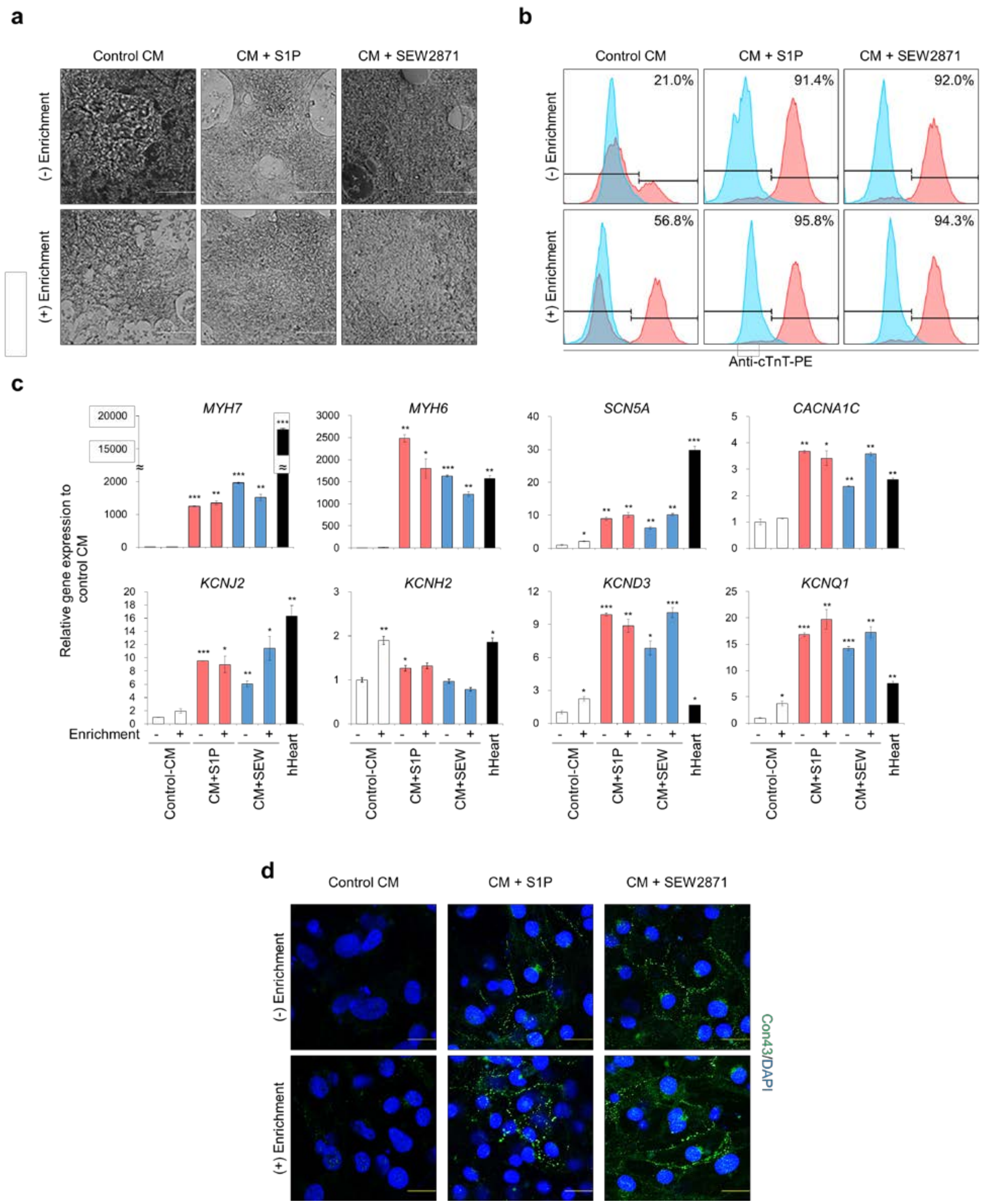

Supplementary Fig. 3. Lactate enrichment of S1P receptor (S1PR)1 activated-CMs. a Bright-field images representing morphologies of control CMs and S1P- or SEW2871-treated CMs with

or without metabolic restriction by lactate enrichment. Scale bar, 200 μm. **b** Flow cytometry histograms of cTnT-positive cell populations in human-induced pluripotent stem cell (hiPSC)-derived CMs following lactate enrichment. **c** Quantitative polymerase chain reaction data related to mature CM markers, including mature atrial, ventricular CM markers, and various ion channel markers in hiPSC-derived CMs. **d** Immunofluorescence analysis showing the expression of CX43 in control and either S1P- or SEW2871-treated CMs. Scale bar, 20 μm. Data are presented as the mean ± SEM (n = 3). * $p < 0.05$, ** $p < 0.01$, *** $p < 0.001$ using a two-tailed *t*-test. CM, cardiomyocyte; S1P, sphingosine-1-phosphate; cTnT, cardiac troponin T; DAPI, 4',6-diamidino-2-phenylindole; CX43, connexin 43.

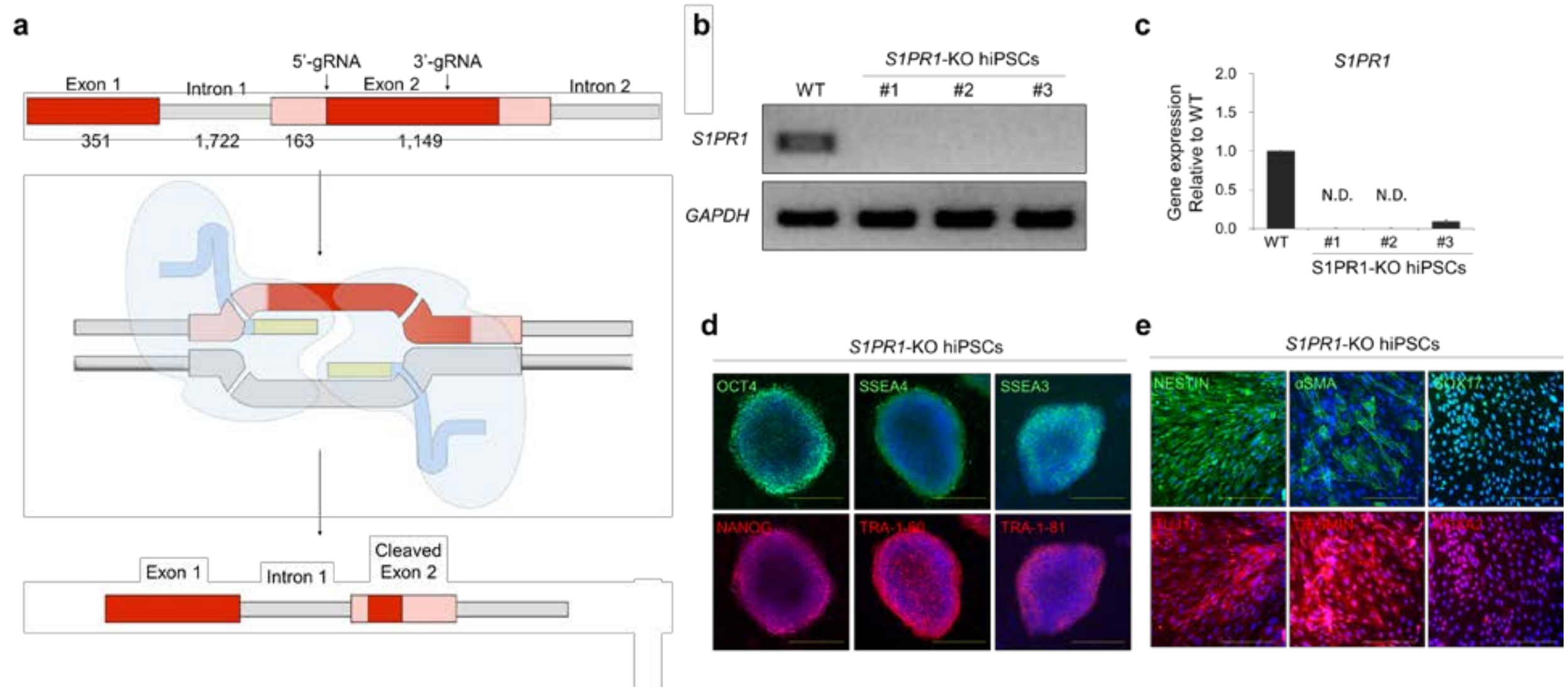

Supplementary Fig. 4. Generation of S1PR1-KO hiPSC lines. a Schematic diagram of clustered regularly interspaced short palindromic repeats (CRISPR)/CRISPR-associated protein 9 gene editing system-based knock-out of *S1PR1*. **b** Polymerase chain reaction analysis for confirmation of S1PR1-KO in three hiPSC lines. **c** Relative *S1PR1* expression in S1PR1-KO hiPSCs. Control hiPSCs were used for positive control. (d–e) Immunofluorescence images of pluripotency markers **d**, and three germ layer differentiation markers **e**. Scale bar, 100 μm. Data are presented as the mean ± standard error of the mean. gRNA, guide RNA; WT, wild-type; S1PR, sphingosine-1-phosphate receptor; KO, knockout; hiPSC, human induced pluripotent stem cell.

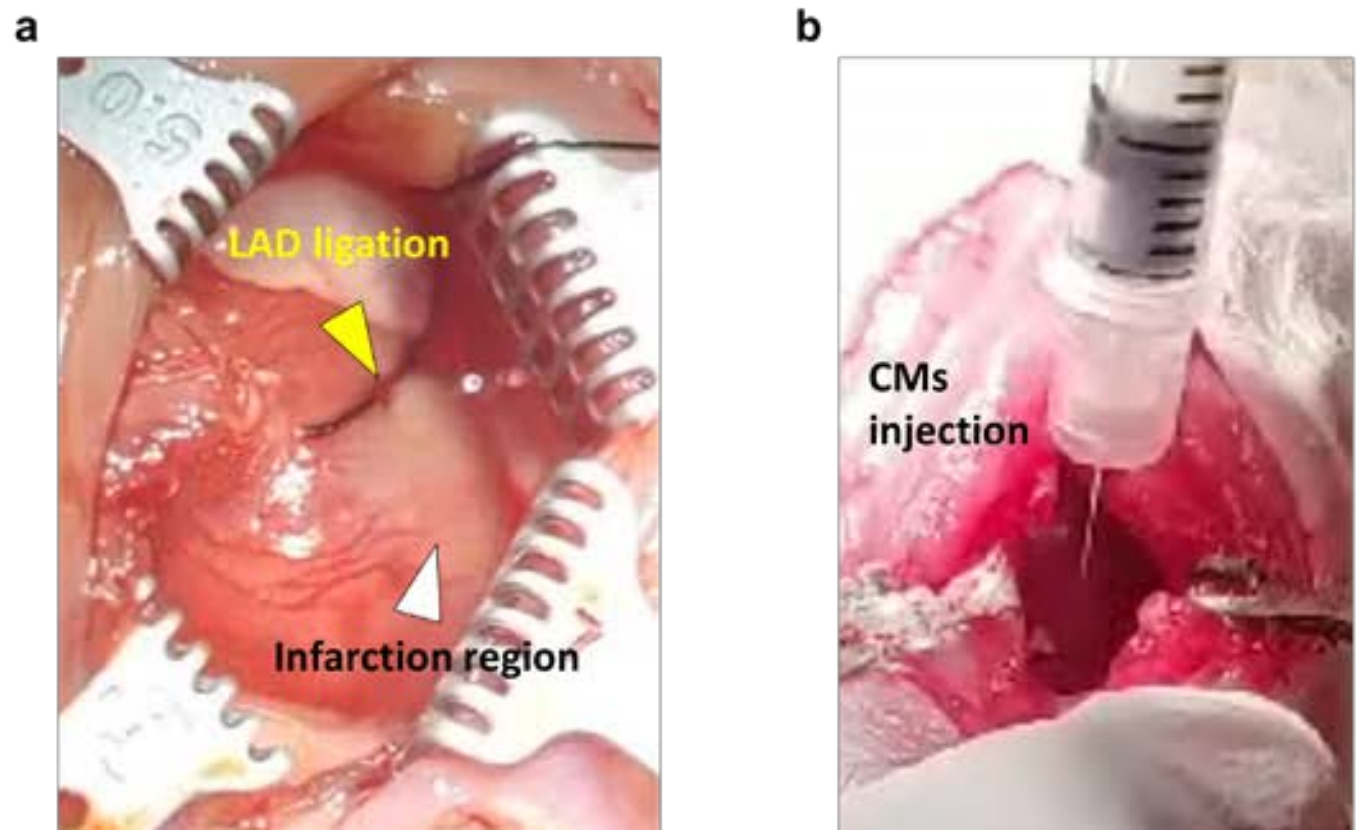

Supplementary Fig. 5. In vivo modeling of myocardial infarction (MI) and therapeutic CM injection. a Images of the LAD artery ligation surgery and MI region. **b** Image of CM injection. LAD, left anterior descending; CM, cardiomyocyte.

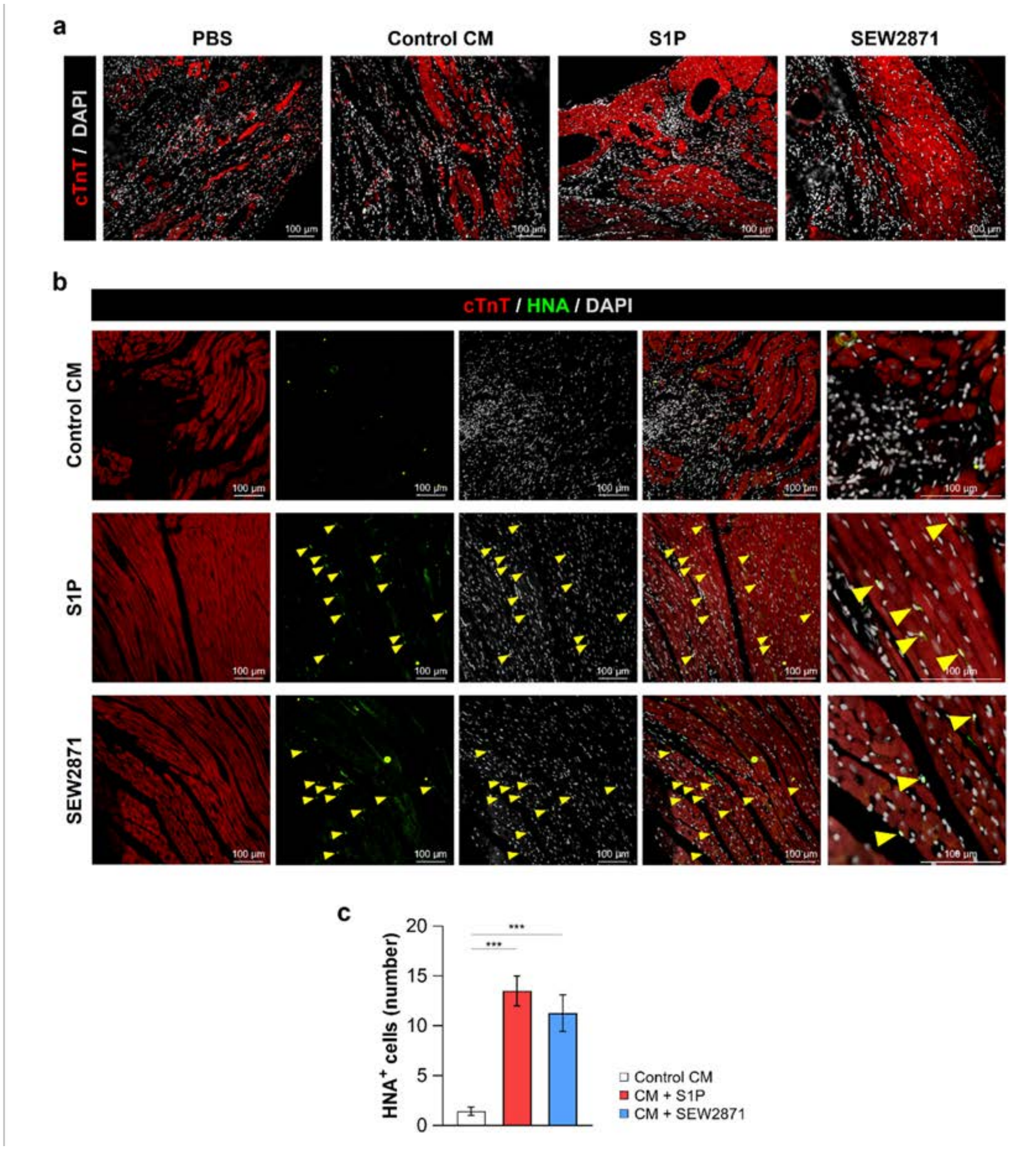

Supplementary Fig. 6. Validation of transplanted CMs in the infarct region. a Comparison of myocardium area (cTnT$^+$ region) within the infarct region among groups at 28 d after MI induction. **b** Representative immunofluorescence images showing co-staining for cTnT and HNA to verify the engraftment of control CMs and S1P- and SEW2871-treated CMs. Arrowheads indicate HNA$^+$ cells within the cTnT$^+$ myocardium region. **c** Quantification of HNA$^+$ cells within the myocardium per image field from three rats per group (n = 6-7). Scale bar, 100 μm. Data are presented as mean

± standard error of the mean ($^{*}p < 0.05$, $^{**}p < 0.01$, and $^{***}p < 0.001$, comparison with control CM).

HNA, human nucelar antigien.

Supplementary Tables

Supplementary Table 1. Primer list used in this study

Gene name	Primer (Forward)	Primer (Reverse)
GAPDH	GAAGGTGAAGGTCGGAGTC	GAAGATGGTGATGGGATTTC
GATA4	GCCTCTCGGTGTGACGAGT	AGCATTGAGCAAAGGGCTCTA
NKX2.5	CAACTTCGTGAACTTCGGCG	GGACTCAGGGTCATGTTGGG
HAND2	GACCCAGGACTCCGGAAAAG	ACGGGAGTGTCCTCTTCGTA
MYH6	ACCTGTCCAAGTTCCGCAAG	AGGTTGGCAAGAGTGAGGTT
MYL7	GGAGTTCAAAGAAGCCTTCAGC	AAAGAGCGTGAGGAAGACGG
MYL2	AGGCGGAGAGGTTTTCCAAG	GGACCACTCTGCAAAGACGA
MYH7	CAGGACCTGGTAGACAAGCTG	ATTCAAGCCCTTCGTGCCAAT
S1PR1	TGACCAACAAGGAGATGCGT	CCTTCGTCTTTCTGGGGGTG
S1PR2	GTCATCTACACGTGGCGCAG	TCCAGAAACGTGGGTGACG
S1PR3	GTGAAGTCCAGCAGCCGTAA	CAGCCAACACGATGAACCAC
S1PR4	CATCATGGGCCTCTATGGGG	AGAGGTTGGAGCCAAAGACG
S1PR5	TCATCTACACGCTCACCAACC	GCTGAAGCTCCCATCAAGGC
SCN5A	TCATCGTAGACGTCTCTCTGGT	GGCTCTTGTTGTTCACGATGGT
CACNA1C	AAGGCTACCTGGATTGGATCA	GCCACGTTTTCGGTGTTGAC
KCNJ2	TGTTGGGTTTGACAGTGGAA	CCCACAGGATTTCATTTGCT
KCNH2	CATTGGCTCCCTCATGTATGCT	GCGTGCTGGAAGTACTCCTCG
KCND3	AGAGAGCTGATAAACGCAGG	CAGGCAGTGCAGCAGGTGAT
KCNQ1	GCGCGGAAGCCTTACGATGT	CAGCTGCGTCACCTTGTCTTC
$MYH6^{Cre}$	TCTATTGCACACAGCAATCCA	CCAGCATTGTGAGAACAAGG
Cleaved S1PR1	GAGCGGAGGAAGTTAAAAGT	GATCCTAAGGCAATGTCCTAG AATGGGACA

Supplementary Table 2. Antibody list used in this study

Antibodies	Company	Catalog No.	Dilution
anti-cTnT-PE	*BD*	564767	1:40
anti-MLC2a	*Synaptic*	311011	1:50
anti-MLC2v	proteintech	10906-1-AP	1:100
anti-cTnT	Abcam	ab8295	1:200
anti-cTnT	Abcam	ab91605	1:200
anti-SAA	Abcam	ab9465	1:50
anti-Con43	Abcam	ab11370	1:100
anti-total p38	CST	8690T	1:1000
anti-total p38	CST	9212S	1:1000
anti-phospho-p38	CST	9211S	1:1000
anti-phospho-p38	CST	9211S	1:1000
anti-total-HSP27	CST	2402S	1:1000
anti-phospho-HSP27	CST	9709S	1:1000
anti-total ERK	CST	4695T	1:1000
anti-phospho-ERK	CST	4370T	1:1000
anti-beta tubulin	Abcam	ab119100	1:1000

Supplementary video information

Supplementary Video 1. Spontaneously Beating Cardiomyocytes Derived from hPSCs

(Separate file) Bright-field movie showing CM morphologies after 14 d of differentiation from hPSCs. Scale bar, 200 μm

Supplementary Video 2. Beating Cardiomyocytes with or without lactate enrichment

(Separate file) Bright-field movie showing control, S1P, or SEW2871-treated CM morphologies after or without lactate enrichment. Scale bar, 200 μm

Supplementary Video 3. Anisomycin-treated Cardiomyocytes derived from S1PR1 KO hPSCs

(Separate file) Bright-field movie showing control, S1P-treated CM morphologies with or without Anisomycin treatment. Scale bar, 200 μm
